# Successful Interventions to Improve Pediatric Vaccine Uptake in Hesitant Cohorts: A Scoping Review

**DOI:** 10.7759/cureus.84399

**Published:** 2025-05-19

**Authors:** Kristen Sibson, Abbigail Shrontz, Jordan Jones, Olivia Hooks, Maria Gerges, Jessica Forbes, Michelle Faliv, Shaima Arshad, Michelle K Knecht, Adam T Wyatt, Robert Casey, Parvathi Perumareddi

**Affiliations:** 1 Pediatrics, Florida Atlantic University Charles E. Schmidt College of Medicine, Boca Raton, USA; 2 Population Health and Social Medicine, Florida Atlantic University Charles E. Schmidt College of Medicine, Boca Raton, USA; 3 Pediatrics, Joe DiMaggio Children's Hospital, Hollywood, USA; 4 Family Medicine, Florida Atlantic University Charles E. Schmidt College of Medicine, Boca Raton, USA

**Keywords:** childhood immunization, parental attitudes, parent education, patient provider communication, pediatric vaccines, undergraduate medical education, vaccine hesitancy

## Abstract

Vaccine hesitancy is a multi-faceted topic that encompasses many different etiologies and solutions. There remains limited literature regarding effective interventions to target these hesitant cohorts. The objective of our paper was to review existing publications that quantitatively measure if tested interventions resulted in an increase in pediatric vaccine uptake within populations that have been evaluated for vaccine hesitancy. Articles were obtained by performing a database search of PubMed, Embase, and Cochrane databases, as well as through hand-searching. Eligible studies included those published in or after 2013, conducted in the United States on pediatric participants eighteen years or younger, and measured quantitatively the success of an intervention. Eight reviewers screened titles, abstracts, and full texts for inclusion. Rayyan facilitated an organized screening process. Data was hand-extracted and collated into charts to identify trends from the seven final articles. Seven articles describing successful interventions that addressed parental vaccine hesitancy and increased pediatric vaccination rates were analyzed. The most prevalent theme among the interventions was healthcare provider communication (71%), followed by parent education (57%), multi-level interventions (57%), standardization of the vaccination process (43%), and healthcare provider education (23%). There were limitations to this scoping review. While vaccine hesitancy was surveyed pre-intervention in each study, those individuals identified as hesitant were not isolated for the target intervention, apart from one article. Additionally, there were different study outcome measures, which made the statistics not directly comparable. Nonetheless, this scoping review identified recurrent evidence-based themes to increase vaccine uptake in hesitant pediatric populations. These interventions should be implemented in medical education and clinical practices to improve pediatric vaccine coverage.

## Introduction and background

The World Health Organization (WHO) acknowledged vaccine hesitancy as a major threat to global health [1]. Despite numerous efforts to improve childhood immunization in the United States, vaccine administration has plateaued over the last 10 years [2], partly due to various barriers and parents’ hesitancy to have their children vaccinated. Some barriers to immunization include a lack of transportation, economic hardship, insufficient clinic hours, and inadequate health care access [3,4]. While overlap of barriers exists, many socioeconomic barriers have direct solutions. Vaccine hesitancy, however, is a more complex issue as it encompasses parents’ attitudes and perspectives. Reasons for vaccine hesitancy include, but are not limited to, cultural or religious beliefs, mistrust in science, relative potential misperception of harm, and lack of knowledge [1,5].

The consequences of delayed or absent immunizations can be devastating [6]. In 1998, a report in The Lancet led to widespread distrust regarding the safety of the Measles, Mumps, and Rubella (MMR) vaccine [6]. The 2019 measles outbreak displays the effects of this distrust; it was the largest recorded in the United States in over three decades, with 1,274 individual cases across 31 different states [7]. Of these cases, 89% of patients had not received the MMR vaccine or had an unknown vaccination status [8]. Additionally, the COVID-19 pandemic sparked a new trend of declining immunization coverage worldwide. This trend led to the largest unremitting reduction in global pediatric vaccination rates in 30 years [9]. According to the WHO, 25 million children globally did not receive their routine immunizations in 2021, which could lead to numerous outbreaks and deaths from vaccine-preventable diseases in the future [9].

Various interventions to combat vaccine hesitancy have been described in the literature. Interventions commonly target the parent, physician, or population and include reminder systems, educational materials, social marketing, and laws related to immunization policy [10,11]. Previous systematic literature reviews have included single and multi-level interventions to increase pediatric vaccine uptake [12-14]. However, these reviews assessed the efficacy of these interventions in participants with no stratification or measurement of vaccine hesitancy. As a result, there is a gap in the literature in understanding which interventions are truly effective in populations inherently more resistant to standard vaccination efforts. Therefore, our study addresses this limitation by focusing on interventions with confirmed vaccine-hesitant participants. By narrowing the scope to hesitant populations, this review provides more precise insights into strategies that successfully increase vaccine uptake within these groups. This approach enhances the clinical applicability of our findings and equips pediatric providers with evidence-based techniques tailored to those who may be averse to vaccines. Recognizing this gap in the literature, we conducted a scoping review to systematically analyze and describe what tested interventions led to a quantitative increase in pediatric vaccine uptake in populations with confirmed vaccine hesitancy.

## Review

Methods

Search Strategy and Eligibility Criteria

The protocol for this review was based on the Arksey & O’Malley framework and the Preferred Reporting Items for Systematic Reviews and Meta-analyses (PRISMA) extension for scoping reviews (PRISMA-ScR) [15,16]. The eight team members (KS, AS, JJ, OH, MG, JF, MF, SA) designed a search strategy and further developed it with a senior medical librarian (MK). PubMed, Embase, and Cochrane databases were utilized to gather articles. Data collection and exportation of citations occurred during July 2023.

The following research question guided our selection: What successful interventions targeting vaccine-hesitant parents or guardians are available for increasing vaccine uptake in pediatric populations in the United States? When considering articles, these definitions were used: (1) Successful Intervention - An intervention that increased vaccine uptake in the pediatric population identified [17-19]; (2) Vaccine Hesitancy (as defined by the SAGE Working Group on Vaccine Hesitancy) - “A delay in acceptance or refusal of vaccination despite access and availability of vaccination services” [20,21]; (3) Pediatric - Children who are eighteen years of age or younger [22,23]; (4) Healthy Children - Children who do not have chronic health conditions or other life-altering circumstances that would affect their eligibility or safety to receive vaccinations [24-27].

Articles were collected by searching the PubMed, Embase, and Cochrane databases with the following keywords and their synonyms, MeSH terms, and Emtree terms: pediatric, parent, vaccine, hesitancy, intervention, and United States. For the Embase search, the following search terms were utilized: (juvenile/exp OR (pediatric OR child* OR adolescent OR newborn OR neonatal OR infant OR juvenile):ab,ti) AND (parent/exp OR caregiver/exp OR (parent* OR caretaker OR ‘care taker’ OR guardian OR caregiver OR ‘care giver’):ab,ti) AND (vaccine/exp OR immunization/exp OR inoculation/exp OR (vaccin* OR inoculation OR immuniz* OR immunis*):ab,ti) AND (‘vaccine hesitancy’/exp OR (hesita* OR refus* OR skepticism OR reluctan* OR delay* OR barriers):ab,ti) AND (‘implementation science’/exp OR (intervention OR implement*):ab,ti) AND (United States/exp OR (‘United States’ OR ‘appalachian region’ OR ‘Confederate States of America’ OR ‘great lakes region’ OR ‘mid-atlantic region’ OR ‘midwestern united states’ OR ‘new england’ OR ‘northwestern united states’ OR ‘pacific states’ OR ‘southeastern united states’ OR ‘southwestern united states’ OR U.S. OR U.S.A. OR ‘United States of America’ OR USA):ab,ti)

The results were exported into Rayyan, an online research tool, to facilitate the screening process by organizing, filtering, and decision tracking in systematic or scoping reviews. While Rayyan automatically identified duplicates, reviewers individually deleted them to verify accuracy. Team members worked in pairs to review the titles and abstracts in a primary screening. Articles that did not meet the established criteria were tagged with specific exclusion reasons, and eligibility disputes among pairs were voted on by the entire team. Articles included after the primary screen underwent a secondary screening, including the addition of three articles retrieved from manual searching. These articles were divided among team members for a full-text review and again tagged with exclusion reasons.

The included studies had successful interventions that quantitatively showed increased pediatric vaccination rates in the United States. These articles were also required to measure parental pre-intervention vaccine hesitancy, include populations of healthy children under eighteen years old, and utilize parent or parent-and-provider-driven intervention(s). Studies performed outside the United States or before January 1st, 2013, were excluded. Additionally, articles were excluded if they were in progress, inconclusive, unsuccessful, unavailable in full-text, or assessed only outcomes of intention to vaccinate, vaccine attitudes, or other qualitative measures. Articles were also excluded if the target population was only health care providers, such as physicians, nurses, medical students, and nursing students.

Data Extraction and Presentation

Data extraction from the final articles included the author(s), publication year, study location, study design, intervention type, comparator, duration of intervention, study population(s), sample size, aims of the study, outcome(s), outcome measurements, methodology, statistical significance, and major findings. Team members were each assigned approximately five full-text articles for data extraction. The research team collectively identified overall themes.

PRISMA

**Figure 1:**
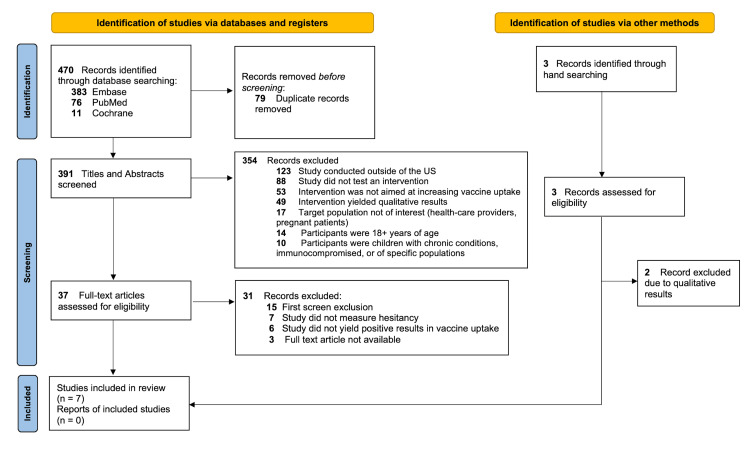
PRISMA Flow Diagram of Study Selection Process

The initial database search resulted in 470 articles from PubMed, Embase, and Cochrane, of which seven met our inclusion criteria (Figure 1). Using Rayyan, 79 duplicate articles were removed, leaving 391 for primary screening. The titles and abstracts of these 391 articles were screened based on inclusion eligibility. Following the primary screening process, 37 articles met our initial criteria and underwent a full-text review in a secondary screening. An additional three articles included in the second screening were found via hand-searching. A total of 40 full-text articles were analyzed for verification of the inclusion and exclusion criteria, and seven remained for the scoping review.

Results

Study Characteristics

A description of the seven studies is provided in Table 1. Of the seven studies, four were completed at clinical practice sites [27-30], individual or small groups of healthcare providers that provide direct patient care (often in outpatient settings), and three were completed at integrated delivery systems [31-33], vertically integrated networks that encompass a broader range of healthcare services (hospitals, post-acute care, multi-specialty group practices). Five studies included participants with children under the age of two [29-33], and two studies included children over the age of nine [27,28]. Two study interventions targeted pregnant mothers and measured outcomes postnatally [31,32].​ 

**Table 1:** Characteristics of Seven Included Studies d: days; DTaP: diphtheria-tetanus-acellular pertussis; HPV: human papillomavirus; mo: months; NA: not applicable; NR: not reported; y: years *Reported for pregnant mothers **Reported for parents of the child a. Integrated delivery system: an organization that owns and operates a network of several healthcare facilities.

Source	Study Type	Sample Size	Vaccine Type	Study Setting	Demographics of Children Vaccinated		
					Age	Sex	Race/Ethnicity
Cox et al., 2022 [27]	Quality improvement	12,270	HPV	2 clinical practice sites	9-13 y	49% female, 51% male	45.2% Hispanic; 34.1% Black, non-Hispanic; 4.4% White; 2% Asian; 14.4% Other
Beck et al., 2021 [28]	Evidence-based practice change model	24	HPV	Clinical practice site	11-17 y	71% female, 29% male	75% White; 21% Hispanic or Latino; 4% Other
Bradshaw et al., 2020 [31]	Quality improvement	21,108	Hepatitis B	Integrated delivery system	0-28 d	*100% female	*67.9% White; 12.1% Asian American; 5.3% African American; 15% Other or refused
Bauer et al., 2021 [29]	Quality improvement	165	Influenza	Clinical practice site	6-24 mo	NR	NR
Glanz et al., 2017 [32]	Randomized control trial	1093	Hepatitis B; rotavirus; DTaP; Haemophilus influenzae type b; pneumococcal conjugate; polio	Integrated delivery system	0-200 d	*100% female	*86.9% White; 12.6% Other
Hofstetter et al., 2017 [30]	Cross-sectional observational study	50	Influenza	8 clinical practice sites	6-19 mo	40% female, 60% male	**77% White; 9% Asian; 7% Black; 7% Other
Opel et al., 2018 [33]	Longitudinal prospective cohort study	73	Hepatitis B; rotavirus; DTaP; Haemophilus influenzae type b; pneumococcal conjugate; polio	Integrated delivery system	0-8 mo	**92% female	**58% White; 6% Asian; 4% Black; 3% American Indian; 3% Pacific Islander; 26% Other

Intervention Characteristics

All articles measured vaccine hesitancy and utilized various interventions to successfully increase the vaccination rate in the study population. A description of the interventions and outcomes for each of the articles is provided in Table 2. 

**Table 2:** Intervention Details and Outcomes from Seven Included Studies HPV: human papillomavirus; PACV: parents’ attitudes about childhood vaccines survey; RCT: randomized control trial; VI: vaccine information; VSM: vaccine information and interactive social media components

Source	Intervention Details	Outcomes
Cox et al., 2022 [27]	Multi-level patient-, provider-, and systems-level intervention. Patient-level: education with handouts and posters. Provider-level: education on motivational interviewing and recommendation language. Systems-level: monthly evening shot clinic, electronic vaccine order set, schedule cards, track patients for reminders and rescheduling.	From 2014 to 2021, HPV vaccine initiation rates increased from 1.1% to 51.8% at age 9, and completion rates increased from 37.4% to 77.2% at age 13. Initiation and completion rates were highest among Hispanic children compared to other races/ethnicities at age 9 (adjusted odds ratio (95% confidence interval) = 1.9 (1.4–2.6)) and at age 13 (OR = 2.3 (1.7–3.0)). When comparing study setting size, the smaller clinic was more successful than the larger one at initiating the first dose at age 9 (64.7% vs 47.5%) and completing the vaccine series at age 13 (85.9% vs 73.6%).
Beck et al., 2021 [28]	PACV survey assessed the degree of parental vaccine hesitancy. Evidence-based practice model utilizing a stepwise approach: 1) Identify population and administer PACV, 2) provide educational material to the parent, 3) strong health care provider vaccine recommendation, 4) targeted individualized decision-making by parent, 5) final parental decision.	In 2019, 100% of the 24 parent participants consented to their child receiving the HPV vaccine compared to the same 6-week period in 2018 when the clinic only had four youth/adolescents vaccinated.
Bradshaw et al. [31], 2020	Standardizing the vaccination process with scripting and timing. Engaging and educating parents. Educating health care providers on strategies to discuss and emphasize the importance of hepatitis B vaccinations.	Of the 21,108 newborns included, hepatitis B vaccination rates administered before hospital discharge increased from 52.4% to 72.5%; rates within 12 hours of life increased from 21.5% to 42.5%.
Bauer et al., 2021 [29]	An open-ended telephone survey was conducted to identify key barriers to influenza immunization. Based on the survey results, a parent-driven quality-improvement program was implemented, which included education, proactive appointment scheduling, and reminders via calls and social media.	Of the 109 children who had not received their influenza vaccine, 100% of their parents were called to remind them to schedule a well-child exam. 78% of these parents responded. Of these, 90% of them attended the appointment, and 99% of the attendees agreed to their child receiving the influenza vaccine. The influenza immunization rate increased from 44% in 2017 to 57% by the end of March 2018.
Glanz et al., 2017 [32]	Participants were randomly assigned to a website with a multidirectional communication model including vaccine information and interactive social media elements (VSM), a website with just vaccine information (VI), or a control group.	When comparing the mean rank of days under-vaccinated, the VSM group was significantly lower than the control (p =.02), but the VI vs controls (p =.08) and the VSM vs VI groups (p =.63) were not statistically different. At age 200 days, the proportions of infants up-to-date with their vaccines in the VSM, VI, and control groups were 92.5, 91.3, and 86.6, respectively. Infants in the VSM group had a greater likelihood of being up-to-date than infants in the control group (odds ratio (95% confidence interval) = 1.92 (1.07–3.47)).
Hofstetter et al., 2017 [30]	PACV survey assessed the degree of parental vaccine hesitancy. Health supervision visits were video recorded and then analyzed using a coding scheme to identify communication behaviors associated with parental vaccine acceptance.	Parental acceptance was higher when providers used presumptive rather than participatory language (94% vs 28%, p<0.001). If parents showed initial resistance, continued pursuit of the original provider recommendation resulted in higher vaccine acceptance than if the provider did not continue pursuing the recommendation (80% vs 13%, p<0.05). Parental acceptance of the influenza vaccine was higher when the provider recommended it in combination with other vaccines rather than separating it (83% vs 33%, p<0.01).
Opel et al., 2018 [33]	Parents reported post-visit whether the physician used presumptive, participatory, or other language styles for discussing vaccines at their child’s 2-, 4-, and 6-month health supervision appointments.	When providers used participatory instead of presumptive language for vaccine recommendations, it led the child to be under-immunized for significantly more days (10.1% more days, p = 0.04, an average of 98 more days for all six recommended vaccines combined).

Major Themes Identified Across Studies

**Figure 2:**
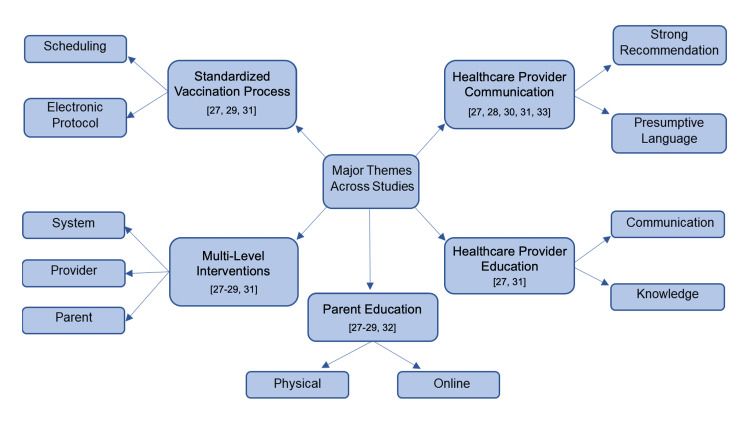
Major Themes [27-33]

The interventions across the seven articles were categorized into five broad themes, as shown in Figure 2. The most common theme observed was healthcare provider communication (5/7 (71%)), followed by parent education (4/7 (57%)), healthcare provider education (2/7 (23%)), and standardization of the vaccination process (3/7 (43%)). Multi-level interventions, consisting of combinations of various themes listed previously, were utilized in most articles (4/7 (57%)). 

Healthcare Provider Communication

Five articles addressed the importance of healthcare provider communication [27,28,30,31,33]. Three studies found that presumptive language increased vaccination rates, as opposed to participatory language [27,30,33]. For example, vaccination was more likely when the provider stated, “We will be administering the second dose of the hepatitis B vaccine today,” instead of asking, “Would you be interested in your child getting their second hepatitis B vaccine today?” Another effective communication style, found in two articles, was a strong provider recommendation [28,30]. One study noted that the stronger a recommendation was, the more likely a vaccination occurred [28]. An additional study found that concurrent vaccine recommendations and a continued pursuit following an initial parent refusal further increased vaccination rates [30].

Three articles allocated dedicated training time to educate providers on a particular communication method to address vaccine hesitancy [27,28,31]. One teaching-based method consisted of a motivational interviewing training session, which urged physicians to engage in nonjudgmental counseling [27]. Another article described training physicians on encouraging an individualized parent decision-making process and addressing concerns and questions about the parents’ hesitancy. The third provided a script and training for nurses and physicians when speaking with hesitant parents [31]. The other two solely observed communication styles or instructed the physicians, without training, to use presumptive language and subsequently monitored the results [30,33].

Parent Education

Four articles included parent education as an intervention target and utilized both physical materials and online platforms to address hesitancy and increase the vaccination rate. The papers that adopted the physical materials approach used basic informational handouts [27,28] and bilingual Spanish and English posters in the office [27,31]. Other studies focused on online education methods. These studies were posted on websites to address safety concerns and misconceptions, as well as general vaccine information [29,31,32], and used social media as a marketing tool [29,32]. One study utilized a website and an interactive social media platform, including a blog, discussion post site, and chat room. Additionally, this study had an anonymous question portal to communicate with an expert [32]. Another intervention combined the physical and online education approach by creating a “Frequently Asked Questions” pamphlet addressing vaccine efficacy and safety. This was posted to a website, Facebook page, and provided to caregivers in the clinic [29].

Healthcare Provider Education

Two articles addressed healthcare provider education as an intervention. These efforts were split into two categories: education on communication strategies and basic vaccine knowledge. Studies addressing education on communication styles were described in detail above [27,28,31]. The studies that aimed to increase provider knowledge focused on teaching about vaccination importance, rates, morbidity and mortality, current guidelines, and safety and efficacy. This was accomplished in several ways, including emails, letters, faculty meetings [27,31], and resident training by attending physicians [27].

Standardized Vaccination Process

Three articles described an interventional component to create a standardized vaccination process [27,29,31]. Two of the three discussed methods for appointment scheduling and reminders via proactive scheduling [29], missed appointment calls [27], and reminders through social media [29], calls [27,29], and cards [27]. Additionally, other practices focused on health care provider team member guidance. Two studies addressed protocol for nurses, including scripts for communication about vaccines [27,31] and standing orders to allow for the recommendation and vaccine administration without physician supervision [27]. A common standardization technique for providers involved automated patient tracking reports, health record order sets [27], and physician note template changes [31].

Multi-level Interventions

Most articles incorporated several of the themes described above to target multiple levels of the health care delivery system, including at the clinic-, provider-, and patient-level [27-29,31]. Two articles included a three-pronged approach: a clinic-wide standardized process for vaccination, education and training of nurses and physicians, and education of parents [27,31]. Other studies adopted a two-level approach. One study focused only on clinic-wide standardization and parent education [29]. Another utilized a unique, stepwise method that first educated clinic staff on pricing options and how to identify candidates for vaccination, and subsequently trained providers on communication methods [28].

Discussion

There are numerous effective interventions in the United States general population, such as parent and patient counseling, maximizing opportunities for vaccination through increased ease of scheduling, offering combination vaccines, and improving accessibility [34]. Given the growing issue of vaccine hesitancy, the objective of this scoping review was to identify successful interventions available for addressing vaccine hesitancy within pediatric populations [1]. While reviewing the literature, there were clear themes observed across the seven included studies - Healthcare Provider Communication, Parent Education, Healthcare Provider Education, Standardized Vaccination Process, and multi-level interventions. Of note, while this scoping review was in progress, the American Academy of Pediatrics (AAP) released a new clinical report in March of 2024 that discusses strategies for improving vaccine uptake that mirror themes discussed in this scoping review, including motivational interviewing and presumptive language for providers, as well as practice-level policies that standardize the vaccination process [35].

Provider communication was the most consistent theme, present in five of the seven articles [27,28,30,31,33]. Clear provider recommendations enhance vaccine acceptance and result in higher perceived susceptibility and severity of disease as well as increased perceived benefit of vaccination [36]. Due to limited literature on targeting only vaccine-hesitant populations, supporting evidence studied a combination of hesitant and/or non-hesitant populations and interventions for vaccine uptake. Strong physician recommendations, according to the recent AAP article, are effective at convincing reluctant parents of the importance of vaccination and were used to increase the likelihood of females receiving HPV vaccinations by four times compared to weaker recommendations [35,37]. Presumptive language, as defined by the 2024 AAP article, is when the physician takes a definitive stance on vaccines by using a closed-ended statement; for example, ”Emily is due for various vaccines today” [35]. This approach differs from the more participatory format, where an open-ended question is used to encourage the parent to share their opinion, such as ”What are your thoughts on vaccines today?” Further research supports the findings of this review that higher vaccination rates are achieved when providers use presumptive versus participatory language [38]. One study showed 73% of parents accepted the HPV vaccine when the provider used presumptive language versus only 22% without [39]. Another successful provider communication intervention is motivational interviewing (MI) [40]. MI is an interviewing approach that helps the provider identify the underlying motivations for the patient’s behaviors [35]. The existing body of literature on MI supports its use in pediatric practices to increase vaccine uptake [35]. One study using MI as an intervention among postpartum mothers showed a statistically significant increase in neonatal vaccine coverage [41]. This information is essential as MI is part of the Undergraduate Medical Education (UME) curriculum and can be incorporated into practice by physicians [42]. The aforementioned physician communication methods are effective tools for enhancing pediatric vaccinations.

Parental education is another tool to address vaccine hesitancy. It allows for misconceptions regarding vaccine safety to be rectified. Some recurrent beliefs held by vaccine-hesitant parents are that vaccines can lead to autism, diabetes mellitus, or developmental delays [43-45] and that vaccines contain toxic chemicals [9]. One of the reasons vaccines are effective at preventing disease is because of the benefits of herd immunity [34], leading to the misconception among some parents that children are not susceptible to diseases covered by herd immunity [46]. This belief has resulted in the resurgence of some vaccine-preventable diseases due to decreasing herd immunity [34]. The recent AAP article highlighted pediatricians as key sources of vaccine information, positively influencing vaccine behavior even among hesitant parents [35]. Future efforts show promise, as studies indicate that graduate medical education (GME) sessions on pediatric vaccines increase residents’ confidence in vaccine discussions [47]. A thorough understanding of vaccine safety monitoring also helps providers address parental concerns [35]. Implementing early education in UME on vaccine safety and communication strategies like MI can further boost medical students’ confidence starting at an earlier stage in training, establishing adequate preparation, and thus improving parent education and adherence in practice later. The use of physical brochures and online platforms can prove helpful to educate parents on such topics to decrease hesitancy and increase vaccine uptake [48]. One study demonstrated that parents who received a handout with information about a vaccine were 9.5% more likely to have their child receive that vaccine [49]. Websites with tailored information about certain vaccines and their misconceptions can increase vaccination in adolescents [50]. Furthermore, interactive educational tools have a greater impact than those that were strictly view-only [32]. However, it is important that the educational information, whether in a brochure or online platform, be presented at a basic level of health literacy and translated into different languages corresponding to the demographics of the area [51]. As the second most common theme found in this scoping review, parental education has proven to be an important intervention to combat vaccine hesitancy.

Provider recommendations are important for parental vaccine acceptance. However, to feel confident giving these strong recommendations, providers must be appropriately educated and trained. When didactic sessions related to pediatric vaccines were implemented in GME settings, residents performed 19% better on knowledge tests, and their comfort levels in discussing those vaccines increased from a rating of 2.9 to 3.76 out of 5.0 [47]. Low confidence due to a lack of knowledge or the ability to answer questions are reasons identified within the literature as barriers to physicians engaging in more conversations about vaccinations [52]. For example, when providers were surveyed about discussions related to HPV vaccinations in males, many did not communicate about the vaccine because they were either uncomfortable discussing the topic or did not understand its importance [53]. Therefore, it is crucial to teach providers to effectively address patients' questions and concerns. With the complexity of pediatric vaccination schedules, it can be difficult for parents to reliably monitor the progress of their child’s immunizations, especially noting that upwards of 22% of children are receiving vaccinations from more than one provider [54]. Through standardization processes such as scheduling protocols, pediatricians can reduce missed opportunities [55]. Reminders implemented into a medical record, standing orders, and text messages provide a behavioral nudge to parents and providers, emphasizing the importance of maintaining proper vaccination scheduling [55-57]. In a 2021 study, Milkman et al. [58] found that sending two text reminders to patients regarding their flu vaccination led to an 11% increase in vaccination rate. Importantly, this intervention proves to be cost-effective, with each text message costing less than a dime [58]. Using standardized scripts ensures providers are using correct terminology, presumptive language, and strong recommendations, but also provides consistency between providers [56,59,60].

Many studies have assessed the benefits of combining the methods described above. In 2017, Chuang et al. [61] stated that the intervention most likely to increase vaccination rates is one that takes a multi-level approach and targets both the healthcare team and the parents. Creating interventions that are aimed at multiple levels leads to more sustained results than those focused on individual levels [62]. Given the numerous reasons for vaccine hesitancy, implementing a multi-level approach can target several rationales simultaneously.

This review highlighted gaps in the literature that need to be addressed to further understand vaccine hesitancy and strategies to combat said hesitancy. Studies that focus solely on a vaccine-hesitant population are noticeably lacking in the literature. A limitation of this scoping review was that in each study, while vaccine hesitancy was surveyed pre-intervention, individuals identified as hesitant were not isolated for the target intervention, apart from one article [33].

One limitation of the evidence of this scoping review was that each study had different outcome measures with no established guidelines for defining a successful intervention. Of the articles, four included p-values to measure significance [27,30,32,33]. In contrast, two measured the percent change in the vaccination rates [29,31], and one measured the total number of vaccinations given pre- and post-intervention [28]. As a result of these different outcome measurements, the statistics between the papers cannot be directly compared, although the interventions used can be analyzed. Therefore, to standardize our criteria, we looked at any quantitative measures that increased vaccination rates.

The artificial intelligence (AI) platform, Rayyan, helped sort through the initial articles. The duplication detection and shared organizational properties were the only AI capabilities utilized. Rayyan allowed the reviewers to keep track of all the articles, assign them to different reviewers, and tag articles with reasons for exclusion. This process was not blinded to other reviewers, leaving room for potential bias. However, each decision had to be supported by reasoning congruent between both reviewers. No article was included or excluded until both reviewers agreed. If the reviewers were at a standstill, the decision was brought to the entire team for discussion. These measures offset some of the potential bias from being unblinded.

Future research is needed to measure the success of an intervention on solely vaccine-hesitant parents. The Parent Attitudes About Childhood Vaccines (PACV) questionnaire is a validated tool with predictive ability to help identify vaccine-hesitant parents, with an observed statistically significant association between a parent’s PACV score and their child’s immunization status [63]. Future studies should utilize this questionnaire in a pre-/post-intervention design to better characterize changes in hesitancy. This approach provides deeper insight into the intervention's impact compared to relying solely on vaccination status as a measure of efficacy. The results of this review can also be applied at an educational level, as UME can incorporate the provider communication strategies into the curriculum. Educating medical students early in their careers on various approaches to combat vaccine hesitancy should provide them with the confidence to engage in these difficult conversations as practicing physicians.

A promising next step is to create a standardized, evidence-based educational tool. Providers and parents can use this personalized tool to enhance their understanding of pertinent information, such as vaccine guidelines, timelines, general facts, and common misconceptions. Once created, the success of this modality would need to be evaluated through randomized controlled trials in hopes of becoming the new gold standard for vaccine education.

## Conclusions

Vaccine hesitancy is a complex topic, and parents may be inclined to delay or refuse a vaccine altogether for their child for numerous reasons, including perceived necessity, relative perception of harm, lack of knowledge, and prior vaccine behavior. Currently, very few studies assess the effect of an intervention on solely vaccine-hesitant parents. This data is critical to understanding what interventions are successful, as reporting what works in a general population likely distorts our understanding of its efficacy, as most parents are vaccine-accepting. Future studies should utilize the validated PACV questionnaire to measure hesitancy and test interventions specifically within vaccine-hesitant parents. Nonetheless, the themes outlined by our scoping review may be effective tools to increase pediatric vaccination uptake among hesitant parents and should be studied further.
